# Cardinal Composition

**DOI:** 10.1007/s10670-022-00591-1

**Published:** 2022-07-29

**Authors:** Lisa Vogt, Jonas Werner

**Affiliations:** 1 Institut für Philosophie, Freie Universität Berlin, Habelschwerdter Allee 30, 14195 Berlin, Germany; 2 Theologische Fakultät, Universität Luzern, Frohburgstrasse 3, 6002 Luzern, Switzerland; 3 LOGOS, Universitat de Barcelona, Carrer de Montalegre 4–8, 08001 Barcelona, Spain; 4 Institut für Philosophie, Universität Bern, Länggassstrasse 49a, 3012 Bern, Switzerland

## Abstract

The thesis of Weak Unrestricted Composition says that every pair of objects has a fusion. This thesis has been argued by Contessa (Analysis 72(3):455–457, 2012) and Smith (Erkenntnis 84(1):41–55, 2019) to be compatible with the world being junky and hence to evade an argument against the necessity of Strong Unrestricted Composition proposed by Bohn (Analysis 69(1):27–31, 2009a, Philos Q 59(235):193–201, 2009b). However, neither Weak Unrestricted Composition alone nor the different variants of it that have been proposed in the literature can provide us with a satisfying answer to the special composition question, or so we will argue. We will then go on to explore an alternative family of purely mereological rules in the vicinity of Weak Unrestricted Composition, Cardinal Composition: A plurality of pairwise non-overlapping objects composes an object iff the objects in the plurality are of cardinality smaller than $\kappa$. As we will show, all the instances for infinite $\kappa$s determine fusion and are compatible with junk, and every instance for a $\kappa > \aleph _0$ is furthermore compatible with gunk and dense chains of parthood.

## Rules of Composition

When does a plurality of objects compose an object? Most philosophers believe that one can answer this special composition question by pointing to certain rules of composition – rules that provide necessary and jointly sufficient conditions for when some objects compose an object, a fusion of them.^1^ A prominent candidate-answer to the special composition question consists in the rule of Strong Unrestricted Composition that says that every plurality of objects has a fusion. However, it has been shown in the recent literature (Bohn 2009a, b; Cotnoir 2014) that this candidate-answer has the feature that it is incompatible with the world being junky, i.e. being such that every object is a proper part. This result has ignited a discussion about weaker rules in the vicinity that are compatible with the world being junky. The aim of this paper is to contribute to this debate by arguing against extant proposals in the literature and exploring a novel family of purely mereological answers to the special composition question. As we will argue, many members of this family are both philosophically satisfying and compatible with gunky objects, the world being junky and dense chains of parthood. Before turning to the topics of gunk, junk and dense chains of parthood below, we will now first clarify what it is for an answer to the special composition question to be purely mereological and what we take it for such an answer to be philosophically satisfying.

A *purely mereological* answer to the special composition question does not take recourse to anything but mereological structure. Everything a purely mereological answer is sensitive to when it comes to determining whether some of them compose a further object are the relations of parthood the objects in a scenario stand in. Below, we will introduce the notion of a mereological model that provides one with a set of objects and a binary relation of parthood defined on the elements of this set. Purely mereological answers are only sensitive to what is represented in such models. Surely, some philosophers believe that the rules of composition are sensitive to more than just their mereological structure. For example, van Inwagen (1990) believes that some objects compose a further object only if the object they compose is a living organism, and Carmichael (2015) holds that whether some objects compose a further object often depends on whether they partake in an event of a special sort. Nevertheless, it is a worthwhile task to investigate whether there are plausible purely mereological candidate-rules available to the friend of junky worlds.

We take it that a *philosophically satisfying* answer to the special composition question is one that is neither trivial nor arbitrary, and that, moreover, *determines* whether some objects have a fusion in a sense to be specified below.

Following Markosian (1998a), we define a trivial answer as one that is an instance of the schema “The *xx* compose an object iff $\phi (xx)$”, where “ $\phi (xx)$” is synonymous to “there is an object composed of the *xx*”.^2^ Concerning arbitrariness, Ted Sider (2001) has argued that a rule of composition is arbitrary if there is “a pair of cases connected by a continuous series such that in one, composition occurs, but in the other, composition does not occur.” (Sider 2001 p. 123). As an example of a continuous series, Sider mentions spatial distance and he upholds that a sharp cut-off point in a continuous series would be metaphysically arbitrary and a “brute fact [that] seems particularly hard to stomach.” (Sider 2001 p. 124). Sider also maintains that every answer to the special composition question except for Strong Unrestricted Composition and nihilism is arbitrary in this sense. We take the considerations in what follows to show that this is not the case.

The core idea behind the demand that a rule of determination should determine fusion is this: In order for it to provide us with a satisfying answer to the special composition question, a rule of composition has to be strong enough: Intuitively, it has to settle *all*, as opposed to only *some* cases of fusion. Below, we will formally make precise what it means to settle all cases of fusion. For now, it will be enough to provide an intuitively necessary condition (that we will show to be telling against extant proposals in the literature): A purely mereological answer to the special composition question determines fusion only if it is not compatible with two worlds that both include nothing but a certain number of simples and fusions of such simples, but one world includes a fusion of all these simples (i.e. a universal object) and the other one does not. The underlying idea is quite simple: When we have a number of simples in a world and there is a purely mereological answer to the special composition question in place, then this answer should at least tell us whether these objects fuse to an universal object.

Concerning the modal status of the rules of composition, the standard position is necessitism, which we identify with the following thesis:

Nec Every rule that governs composition holds with necessity.

Our results are relevant for necessitists, for we have a rule to propose to them that allows them to accommodate both gunk and junk. Some (e.g. Cameron 2007) deny Nec and hold that it is a contingent matter which rules govern composition. We also have something to offer to contingentists who accept that necessarily some mereological rules govern fusion. We can treat these contingentists to an entire family of available rules, as will become clear in Sect. 3.

In what follows, we will assume that the relation of parthood forms a partial ordering relation (i.e., it is a reflexive, transitive and antisymmetric relation), and that the axiom of Strong Supplementation holds.^3^ This amounts to assuming that mereology is extensional, i.e. that no two objects have the same proper parts. Although it would be an interesting question which of the considerations of the paper could be redeemed within an intensionalist setting, for reasons of space, we have to leave this discussion for another occasion.

We will say that *y* is the *fusion*, or *sum*, of the *xx* iff *y* overlaps all and only those objects that overlap one of the *xx*.^4^ Given this notion of fusion, we may define a notion of *overlap-equivalence* between pluralities of objects, according to which two pluralities *xx* and *yy* of objects are equivalent iff the following holds: An object overlaps one of the *xx* iff it overlaps one of the *yy*. Fusion can then be conceived of as a case of overlap-equivalence that is singular on one side: An object *y* is the fusion of a plurality *xx* iff *y* is overlap-equivalent to the *xx*. Following van Inwagen (1990) p. 29, we will say that some objects *compose* an object iff they do not pairwise overlap and some object is the fusion of them.^5^

On a first glance, given that composition concerns exclusively not pairwise-overlapping objects, rules of composition would seem to provide us only with a partial answer to the question of when an arbitrary—overlapping or non-overlapping—plurality of objects has a fusion. Luckily, however, the following result can be shown to hold:

Connection: For every plurality of objects *yy*, there is a plurality of pairwise non-overlapping objects *xx* that are parts of the *yy* such that *xx* and *yy* are overlap-equivalent.^6^

Given that overlap-equivalent pluralities have the same fusions, in order to determine whether a given plurality of objects has a fusion, it suffices to consider an overlap-equivalent plurality instead. The principle of Connection now guarantees that, for any given plurality, we will find one whose members do not pairwise overlap – and hence, one to which we can apply rules of composition.

In this way, Connection coordinates between fusion and composition, and allows us to move back and forth between the two. We will prove Connection in the appendix of this paper.

## Strong and Weak Unrestricted Composition

After having set out the necessary background on rules of composition, we now turn to the topic of junky worlds. Bohn (2009a) has objected to the necessity of Strong Unrestricted Composition with an argument based on the following two premises:

(A) Possibly, every object is a proper part of some object.
(B) Necessarily, if Strong Unrestricted Composition holds, then there is an object that is not a proper part of some object.

Premise (A) says that the world might have turned out to be junky, i.e. such that every object is a proper part. Together with premise (B), it yields the result that the rule of Strong Unrestricted Composition does not hold with necessity. In this paper, we will accept premise (A) without argument.^7^ The question we address in this paper is which necessary rules can determine composition, given that junky worlds are possible. Premise (B) can be proven to hold if the principle of Weak Supplementation, the principle that if *x* is a proper part of *y*, then some part of *y* does not overlap *x*, holds.^8^ By the definition of fusion, every fusion of all objects overlaps every object. From Weak Supplementation it follows that no object can be a proper part of some object, unless there is an object it does not overlap. Therefore, no object has a fusion of all objects as a proper part.

Defenders of unrestricted composition who wish to accept the possibility of the world being junky have reacted to this argument by replacing the thesis of Strong Unrestricted Composition with the thesis of Weak Unrestricted Composition that says that every pair – rather than every plurality – of objects has a fusion.^9^

As it stands, however, the rule of Weak Unrestricted Composition clearly fails to provide an answer to the special composition question: It merely yields a sufficient, but not a necessary and sufficient condition for when a plurality of objects has a fusion. And it is obviously also a non-starter to hold that all *and only* pairs of objects have a fusion. For this overly restrictive rule would preclude the possibility of any world with at least three distinct objects *x*, *y*, *z*. According to the given rule, *x* and *y* would have a fusion, [*x*, *y*], and there would be a fusion of [*x*, *y*] and *z*, [*x*, *y*, *z*]. At the same time, the rule would yield the result that [*x*, *y*, *z*] does not exist, for it is the fusion of more than two objects.

In the literature, one can find two candidates for rules that entail Weak Unrestricted Composition, are weaker than Strong Unrestricted Composition, and provide an answer to the special composition question. In what follows, we shall argue, however, that they both have undesirable features and thus fail to yield convincing answers.

The first candidate has it that all and only *finite* pluralities of objects have a fusion, and is discussed in Bohn (2009a). This rule has some *prima facie* plausibility, for it pays justice to the idea that every object is constructed by finitely many applications of pairwise fusion. To see that it is compatible with the world being junky, consider a world $w_{a}$ which contains nothing but countably infinitely many simples and fusions thereof, in which the given rule applies, and mereology is extensional.

However, this candidate rule has the undesirable feature of ruling out the existence of gunky objects, i.e., objects that have proper parts and that are such that every proper part of them has a proper part:^10^ In every gunky object, one can find an infinitely descending chain of proper parthood. And given the principle of Weak Supplementation, every object from which an infinitely descending chain of proper parthood descends has infinitely many non-overlapping proper parts.^11^ Given that the motivation for accepting the possibility of gunky objects is at least as strong as the motivation for accepting that the world is possibly junky, the present candidate-answer to the special composition question does not constitute much progress. We should continue to look for an answer that allows for both gunk and junk.

The second candidate consists in adding the following rule to Weak Unrestricted Composition: Every object that is not a simple is the fusion of exactly two further objects. We will call this rule Weak Unrestricted Splitting. This idea has been proposed in the literature by Smith (2019).^12^ If Weak Unrestricted Composition is combined with Weak Unrestricted Splitting, one arrives at the following answer to the special composition question: A plurality of objects *xx* have a fusion iff there is only one *xx*, or there are two objects $y_{1}$ and $y_{2}$ such that $y_{1}$ and $y_{2}$ are jointly overlap-equivalent to the *xx*.^13^

An answer to the special composition question that combines Weak Unrestricted Composition with Weak Unrestricted Splitting does not rule out either gunky objects or that the world is junky. For a world that validates the two combined rules and is junky, it suffices to consider once again our world $w_{a}$.^14^ Moreover, gunky objects are also compatible with the given combination of rules, as long as the gunky object is such that every part of it can be decomposed into two further parts.

We argue against the combination of Weak Unrestricted Composition with Weak Unrestricted Splitting not by showing that it rules out scenarios that should not be ruled out, but by showing that it fails to determine fusion (and hence to be philosophically satisfying in the sense introduced above). Recall that a purely mereological answer to the special composition question determines fusion only if it is not compatible with two worlds which both include nothing but a certain given number of simples and fusions of these simples, but one world includes a fusion of all the simples (i.e. a universal object) and the other one does not.

The combination of Weak Unrestricted Composition and Weak Unrestricted Splitting, however, allows for the existence of two such worlds: For a world without a universal object, on the one hand, consider the already introduced junky world $w_{a}$. For a world with a universal object, on the other hand, consider a world $w_{b}$ in which there is nothing but countably infinitely many simples and fusions of them, and mereology is classical, i.e. extensional and such that Strong Unrestricted Composition holds. Strong Unrestricted Composition entails Weak Unrestricted Composition, and $w_{b}$ clearly also validates Weak Unrestricted Splitting. Consequently, both $w_{a}$ and $w_{b}$ validate Weak Unrestricted Composition and Weak Unrestricted Splitting, and in both $w_{a}$ and $w_{b}$ every object is a simple or the fusion of at most countably infinitely many simples. At the same time, in $w_{a}$ there is no universal object and in $w_{b}$ there is a universal object. This allows us to apply our necessary criterion for rules determining fusion and shows that the combination of Weak Unrestricted Composition and Weak Unrestricted Splitting fails to determine fusion.

This has a noteworthy consequence for those philosophers who not only hold Nec (the thesis that rules of composition hold with necessity), but also subscribe to the following thesis:

Det Necessarily, a purely mereological rule determines fusion.

This thesis says that every possible case of fusion is determined by a purely mereological rule. The combination of Nec and Det is incompatible with:

P-w_a_/w_b_: Both $w_a$ and $w_b$ are possible.

If every case of composition is determined by a rule that holds with necessity, then either countably infinitely many simples have a fusion in every world, or in no world.

What is the general idea behind the necessary criterion just used? Informally, some rules of composition can be said to provide a satisfying answer to the special composition question only if the following holds: The rules of composition have to settle not only some, but rather, *all* cases of composition in order to provide a satisfying answer to the special composition question.

Remember that the necessary condition on what it is for rules to determine fusion we used so far is based on the idea that, in an atomistic world, the rules of composition cannot determine fusion if they are silent about whether there is an universal object composed of all of them. We can generalize in two ways: First, the underlying motivation generalizes to all objects, not only to the universal object. Secondly, it generalises to worlds where not every object is composed of simples.

We wish to capture the idea that if every object in a world is composed of the *xx*, then the rules of composition ensure for every subplurality of the *xx* whether it has a fusion or not. In this way, the rules of composition settle each case of fusion.

To formally spell out the idea of mereological determination, we need the notion of a mereological model. Let $M=\langle D,\sqsubseteq \rangle$ be a model that consists of a set of objects *D* (the domain of *M*) and $\sqsubseteq$, a binary relation on *D*. We take models to represent the mereological structure of a world, with the members of *D* representing all the concrete objects in the world and $\sqsubseteq$ representing the relations of (proper or improper) parthood between them. Given that we assume parthood to be reflexive, transitive and anti-symmetric, we only consider po-models in what follows, i.e. models in which $\sqsubseteq$ is a partial ordering on *D*. In the remainder of the paper, “model” is thus always to be understood as “po-model”.

In what follows, we will abstract away from the particular cases and provide a general criterion that rules of composition have to meet to provide a satisfying answer to the special composition question. To do so, we will think of rules of composition as constraints on admissible mereological models, principles that rule out some models as impossible. We say that *M* is admissible with respect to a set of mereological rules iff it respects these rules.

In our formal definition of mereological determination, we employ the notion of a submodel. As is common, we say that a model $M_{1}=\langle D_{1},\sqsubseteq _{1}\rangle$ is a *submodel* of $D_{2}=\langle D_{2},\sqsubseteq _{2}\rangle$ just in case $D_{1}\subseteq D_{2}$ and $\sqsubseteq _{1}$ is the restriction of $\sqsubseteq _{2}$ on $D_{1}$, i.e. such that $x\sqsubseteq _{1} y$ iff $x \sqsubseteq _{2} y$ and $x,y\in D_{1}$.

Next, we define the notion of a base of objects, i.e., a set of objects that allows us to fuse every object in a given model: A model $M_{1}$ is a *base* for another model $M_{2}$ iff $M_{1}$ is a submodel of $M_{2}$ such that every object in $D_{2}$ is the fusion (with regard to $M_{2}$) of objects in $D_{1}$.^15^

Now we can render precise what it is for a rule to determine fusion: A rule of composition *determines fusion* iff, for all models $M_{1}, M_{2}$ that are admissible with regard to this rule: If $M_{1}$ is a base for $M_{2}$, then $M_{1} = M_{2}$.

To see how this definition works out in the case of Weak Unrestricted Composition and Splitting, note that, clearly, every model for a world like $w_{a}$ can be a base for a model for a world like $w_{b}$, and yet the models are not identical.

Note that the idea of mereological determination has the idea of an order from the composing objects to the composed objects built in. This pays justice to the fact that rules of composition are (as the name suggests) supposed to govern the relation of composition, a relation that brings us from parts to the wholes they compose.

To see that the demand for determination of fusion has also a broader application, consider e.g. the following rule of Moderately Unrestricted Fusion, a proposal in the vicinity of Weak Unrestricted Composition due to Bostock (1979): If there is an object *y* such that all of the *xx* are parts of *y*, then the *xx* have a fusion *z*. Now, in extensional mereology, if *z* is the fusion of the *xx*, then all of the *xx* are parts of *z*. Hence, the right-to-left direction of Moderately Unrestricted Fusion is guaranteed to hold, and the principle can be strengthened to a biconditional, thus yielding a rule of composition in our sense. This rule, however, would likewise fail to determine fusion. To see this, note that the rule would be e.g. compatible with both a model $M_{1}$ that contains only two simples *x* and *y*, and a model $M_{2}$ that contains *x* and *y* and a fusion *z* of them. Clearly, $M_{1}$ is a base for $M_{2}$, and yet $M_{1} \ne M_{2}$.

We have seen that Weak Unrestricted Composition and Weak Unrestricted Splitting together are too weak to determine fusion. This observation motivates the question which rules that entail Weak Unrestricted Composition, but not Strong Unrestricted Composition, do so. In the remainder of this paper, we will now suggest and further explore a variety of such rules.

## Cardinal Composition and Countable Composition

Our proposal consists in the following rule-schema of Cardinal Composition:

- Cardinal Composition (CC): The *xx* compose an object iff the *xx* are pairwise non-overlapping and of cardinality smaller than $\kappa$.

CC yields a rule of composition for every cardinal number $\kappa$. We will designate the corresponding instance for $\kappa$ with ‘Cardinal Composition_κ_’/ ‘$\hbox {CC}_\kappa$’. As we will discuss in short, necessitists will have to pick one instance of CC. And also contingentists have good reasons to not accept CC in full generality, but restrict it to specific kinds of cardinals.

$\hbox {CC}_1$ is incompatible with there being any objects whatsoever. If a world contains at least one object, the left-to-right direction of $\hbox {CC}_1$ sets the unfulfillable demand that the plurality consisting solely of this object has a cardinality smaller than 1. $\hbox {CC}_2$ is simply the well-known rule of compositional nihilism. In those cases in which $\kappa$ is larger than 2, but still finite, $\hbox {CC}_\kappa$ precludes the existence of more than $\kappa$-many pairwise non-overlapping objects, for reasons analogous to those discussed in the context of the rule that all and only pairs of objects have a fusion. The problem is this: If $\kappa$ is larger than two but still finite, fusing less than $\kappa$ many objects which individually have less than $\kappa$ many non-overlapping parts can result in an object that does not have less than $\kappa$ many parts. And then, $CC_\kappa$ would on the one hand license the existence of each of the individual objects and demand that there be a fusion of them, but on the other hand conflict with the existence of the resulting fused object.

As we will show in the appendix, this problem arises more generally if and only if $\kappa$ is a so-called *singular*, as opposed to *regular* cardinal. A cardinal $\kappa$ is regular iff it has a *cofinality* that equals $\kappa$. The cofinality of $\kappa$ is the smallest cardinal $\lambda$ such that some union of $\lambda$ many sets of cardinality less than $\kappa$ has the cardinality $\kappa$. A cardinal $\kappa$ is singular iff it is not regular, that is, iff it either has no cofinality or a cofinality that does not equal $\kappa$.

A couple of examples might help to clarify the definition of regularity. Among the non-zero finite cardinals, 2 is the only regular cardinal.^16^ 1 is singular since 1 has no cofinality. And every finite cardinal larger than 2 is singular because it has 2, and thus a cardinal smaller than itself as its cofinality.^17^ If you take any finite number of sets, each of which has finitely many members, then the union of this set also has finitely many members. It takes the union of countably infinitely many sets of finite cardinality to arrive at an infinite cardinality. Hence, the cofinality of the first infinite cardinal $\aleph _{0}$ (the limit-cardinal of the finite cardinals) is $\aleph _0$. This makes $\aleph _{0}$ a regular cardinal. The limit-cardinal $\aleph _{\omega }$, by contrast, is an example of a singular cardinal. It is defined as $\bigcup _{n \in {\mathbb {N}}} \aleph _{n}$. For any $n \in {\mathbb {N}}$, $\aleph _{n} < \aleph _{\omega }$ and $| {\mathbb {N}} | = \aleph _0 < \aleph _{\omega }$. $\aleph _{\omega }$ is thus the union of $\aleph _0$ many (and hence less than $\aleph _{\omega }$ many) sets each of which has a cardinality smaller than $\aleph _{\omega }$. And in consequence, the cofinality of $\aleph _{\omega }$ is smaller than $\aleph _{\omega }$, making $\aleph _{\omega }$ a singular cardinal.

Since problems with CC arise if and only if $\kappa$ is singular, we will put the instances of CC for singular $\kappa$s to the side and restrict attention to the instances where $\kappa$ is regular. In what follows, we will thus take ‘$\kappa$’ to range over regular cardinals only, and reserve the terms ‘Cardinal Composition/CC’ for the corresponding instances.

Every instance of CC provides an answer to the special composition question.^18^ However, one might also wish to know when an arbitrary – pairwise overlapping or non-overlapping – plurality of objects has a fusion. By invoking Connection, every instance of CC can be shown to be equivalent to the corresponding instance of the following schema, as we will prove in the appendix:

- Cardinal Composition* (CC*): The *xx* have a fusion iff there is a plurality *yy* of parts of the *xx* that are overlap-equivalent to the *xx*, and such that the *yy* are pairwise non-overlapping and of cardinality smaller than $\kappa$.

As advertised above, the instances of CC will be of interest to the contingentist who holds that no rule of composition governs composition in every possible world, but that, in every possible world, *some* rule governs composition. The schema provides them with a stock of purely mereological rules that they might take to hold in some worlds: either in all of them, or at least in some of them.

The necessitist, by contrast, will have to pick an instance of CC that they take to necessarily govern composition. We take the natural candidate to be the instance for $\kappa = \aleph _1$, the successor of the cardinality of the natural numbers. According to this rule – which we call ‘Countable Composition’ – a plurality of pairwise non-overlapping objects compose a further object iff it has at most countable many members:

- Countable Composition: The *xx* compose an object iff the *xx* are pairwise non-overlapping and countably many (i.e., either finitely many or countably infinitely many).

As we will show below, Countable Composition is the most restrictive instance of CC (i.e., the one that yields the ‘fewest’ fusions) that is compatible with both gunk and junk. By contrast, there is no least restrictive instance, given that there is no largest regular cardinal.^19^ In this way, Countable Composition ‘stands out’ among the different instances of CC, thus providing us with some defeasible reason for favoring this instance over others. Moreover, it might be argued that Countable Composition is the only instance of CC that allows for gunk and junk whilst being compatible with the view that every composite object is the result of successive applications of a binary fusion-operation, an idea that seems to stand in the background of Weak Unrestricted Composition (see also Cotnoir 2014 p. 656 on this).

We take the following (somewhat picturesque) consideration to suggest that (i) it is conceptually possible that an object that has countably many pairwise non-overlapping parts is the result of successive applications of a binary fusion-operation and that (ii) objects with uncountably many pairwise non-overlapping parts cannot be generated in this way:

You find an old lady in a hut, the mistress of composition, and she shows you an object that can be decomposed into simples and that she claims to have produced by successively fusing the previous day’s results with a new simple every single day. Astonished, you find out that the object has countably infinitely many parts. Did the old lady lie? Not necessarily, for both the world and the lady might be infinitely old, forever having added a simple to the object for every single day. For every simple you point to, she can tell you how many days ago she added it to the object. An analogous story doesn’t work for an object that is composed of uncountably many simples. If the object the lady shows you has more than countably many parts, then it cannot be the result of successive applications of a binary fusion-operation. For some simple parts of the object, she won’t be able to tell you how many days ago she added it to the object. To see how gunky objects can be conceptualised as the result of successive applications of pairwise fusion, consider the following variant of the story: Today the old lady fused objects $g_{1}$ and $g_2$ to produce gunky object *g*. Yesterday she fused $g_{11}$ and $g_{12}$ to produce $g_1$. Two days ago she fused $g_{21}$ and $g_{22}$ to produce $g_2$. Three days ago she fused $g_{111}$ and $g_{112}$ to produce $g_{11}$ and so on *ad infinitum*.^20^

**Fig. 1:**
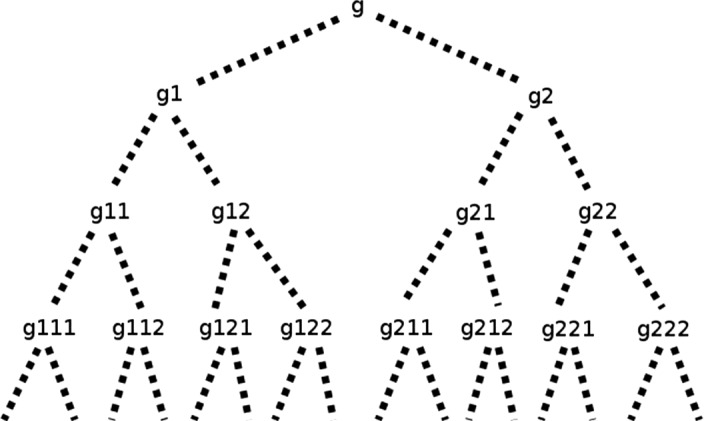
The gunky object *g*

We take a scenario to be conceptually possible iff it does not invoke any logical or conceptual contradiction. Assuming that there are infinitely many past days, an infinitely old lady does not generate a logical or conceptual contradiction. Hence, our story supports the claim that if we wish to countenance every object that can be generated by successive applications of pairwise fusion, then we should countenance all and only those object that are given by Countable Composition.

We take this to suggest that, from a mereological point of view, Countable Composition is the natural choice for the necessitist.

That being said, we acknowledge that some necessitist’s independent philosophical commitments might provide reasons to choose a $\kappa$ different from $\aleph _{1}$. Here is an example: According to certain substantivalist views on the nature of spacetime, spacetime points qualify as concrete objects and thus also fall under the scope of rules of composition. Countable Composition would exclude the existence of regions of spacetime with a Lebesgue measure greater than zero, given that such regions would contain uncountably infinitely many spacetime points.^21^ Another example (which we owe to a referee) concerns the relation between objects and the regions they are located at. Countable Composition rules out that any object meets the following two conditions: (i) There is an object located at a region that has uncountably many spacetime-points as subregions and (ii) each subregion of a material object contains a part of that object.^22^ It should also be clear that a defender of Nec who holds that necessarily every case of composition is governed by the rule of Countable Composition cannot accommodate the possibility of the junky world $w_a$. We take the possibilities of $w_a$ and $w_b$ to be equally *prima facie* plausible. And (as already mentioned above) the possibility of both of them (i.e. P-w_a_/w_b_) is incompatible with the combination of Nec and Det. We take this to suggest that there is no perfect rule for the necessitist. The best thing the necessitist can do is to settle for the rule that accommodates the widest array of prima facie possibilites while being relatively simple (and fulfilling further theoretical desiderata, such as being non-arbitrary and determining fusion). We provide them with a stock of candidates and, despite its potential shortcomings, we suggest that Countable Composition might be the best rule for necessitists all things considered.

At this point the reader might worry (and an anonymous referee did) that this makes our proposal inferior to denying Det and replacing it with:

N-WUC/S: Weak Unrestricted Composition and Weak Unrestricted Splitting hold with necessity.

Although we do not have a knock-down-argument against this position, we can offer some considerations that contribute to making this option seem a bit less attractive. Presumably, the conceivability-considerations that speak in favour of the possibility of $w_a$ allow for simples of any kind that stand in any kind of relations (spatial or whatnot) as inhabitants of $w_a$. The same goes for $w_b$. Now, if one wonders for what reasons in $w_b$ the simples compose a universal object and in $w_a$ they do not, one will have to accept that this is just a brute and contingent fact. We wonder why someone inclined to accept brute and contingent cases of composition should be an ardent defender of Nec. Of course, the defender of Nec might stick to their guns and maintain their position irrespective of the costs. But we hope that this consideration might move some philosophers sympathetic to P-w_a_/w_b_ and N-WUC/S to consider the following alternative view.

If one is prepared to give up Nec, then one can have all of P-w_a_/w_b_, Det, and N-WUC/S by subscribing to the following claim:

NCC: Necessarily, there is a regular infinite $\kappa$ such that $\hbox {CC}_\kappa$ governs all cases of composition.

$\hbox {CC}_{\aleph _0}$ is the rule that all and only finite collections of non-overlapping objects have a fusion. This rule is compatible with $w_a$. In contrast, for any regular $\kappa > \aleph _0$, the rule $\hbox {CC}_\kappa$ has it that in a world that contains just countably many simples and fusions thereof, every plurality of objects has a fusion. This makes NCC compatible with $w_b$. Moreover, NCC clearly entails Det and Weak Unrestricted Composition. Finally, that every instance of $\hbox {CC}_\kappa$ entails Weak Unrestricted Splitting will be shown in the appendix (Theorem 6). We contend that these properties make adopting NCC an attractive option for the friend of P-w_a_/w_b_ and N-WUC/S.

Although none of our proposals will convince the ardent defender of Nec and P-w_a_/w_b_, our paper will also be of interest to them: First, we developed and made precise the notion of fusion-determination, which gives us a novel criterion for evaluating rules of composition. This allows us to clarify an important distinction between rules like Strong Unrestricted Composition (any *xx* whatsoever have a fusion) and the combination of Weak Unrestricted Splitting and Weak Unrestricted Composition. Second, our paper answers the question whether there are purely mereological positions that are weaker than Strong Unrestricted Composition and stronger than the combination of Weak Unrestricted Splitting and Weak Unrestricted Composition (i.e. rules that entail this combination but are not entailed by it). NCC gives us one such a position.

After these dialectical considerations, we will now go on to show that all the instances of CC have a number of welcome features, and can overcome all the problems that Strong and Weak Unrestricted Composition face. No instance of CC is trivial or arbitrary in the sense specified by Sider. Furthermore, every instance of CC determines fusion, as we will prove in the appendix.

To see that for every infinite $\kappa$, $\hbox {CC}_\kappa$ is compatible with the assumption that the world is junky, take a world $w_{c}$ with an extensional mereology in which there are more than $\kappa$ many simples and $\hbox {CC}_\kappa$ holds. In such a world, for every plurality of $\kappa$ many simples *xx*, there is another plurality of $\kappa$ many simples such that: All objects among the *xx* are among the *yy*, but not vice versa. Since the object composed of the *xx* is a proper part of the object composed of the *yy*, every object is a proper part, i.e., the world is junky.

To see that for every $\kappa > \aleph _0$, $\hbox {CC}_\kappa$ is compatible with a gunky object, consider the gunky object *g* that is composed of $g_{1}$ and $g_{2}$, which in turn are composed of $g_{11},g_{12}$ and $g_{21},g_{22}$, respectively, and so on. The gunky object is such that every part of it is composed of some of the $g_{i}$.^23^

We depart from the observation that such a gunky object is clearly compatible with classical mereology, i.e., the combination of extensional mereology with Strong Unrestricted Composition. If we can show that *g* does not have uncountably many pairwise non-overlapping parts, then this guarantees that it is also compatible with CC, and, more generally, with all instances of CC$\kappa$ that allow for objects having countably infinitely many parts. This can be shown as follows: Every part of *g* is the fusion of some of the $g_{i}$. Let *G* be the set of the $g_{i}$. Let *Par*(*g*) be the set of parts of g. Let *f* be the function from ${\mathcal {P}}(G)\backslash \emptyset$ (the set of non-empty subsets of *G*) to *Par*(*g*) that maps every non-empty subset of *G* to the part of *g* the members of this subset fuse. This function is not injective (e.g. $\lbrace g \rbrace$ and $\lbrace g_{1},g_{2} \rbrace$ are both mapped to *g*), but it is surjective. It is surjective because every part of *g* is the fusion of some $g_{i}$. It is easy to see that every two subsets of *G* that have a member in common are mapped onto two overlapping objects. It follows that for every $S\subseteq {\mathcal {P}}(G)\backslash \emptyset$: The subset of *Par*(*g*) the members of *S* are mapped to is such that its members are pairwise non-overlapping only if the sets in *S* are pairwise disjoint. Given this and the surjectivity of *f*, in order to show that every subset of *Par*(*g*) whose members are pairwise non-overlapping has at most countably many members, it suffices to show that every subset of ${\mathcal {P}}(G)\backslash \emptyset$ whose members are pairwise disjoint has at most countably many members. Every subset of ${\mathcal {P}}(G)\backslash \emptyset$ such that its members are pairwise disjoint is a subset of some partition of *G*. No partition of *G* has more elements than the finest partition of *G* that consists of all and only the singletons of the members of *G*. This partition has as many members as *G*. As can easily be shown by diagonalization, the $g_{i}$ are countably many and hence *G* has countably many members.

Obviously, the existence of a gunky object like *g* is compatible with the existence of more than $\kappa$ many simples (or more than $\kappa$ many mereological duplicates of *g*). In scenarios of this sort, we have a gunky object in a junky world (or even a junky world in which all objects are gunky). The present proposal thus also allows for such *hunky* worlds.^24^

For $\kappa >\aleph _0$, $\hbox {CC}_\kappa$ is also compatible with dense chains of proper parthood, as a further examination of the gunky object *g* shows. Consider two members of *G*, $g_{x}$ and $g_{y}$, such that $g_{x}\sqsubset g_{y}$. Then, by Weak Supplemenation, there will be a $g_{i}\in G$ that does not overlap $g_{x}$ and is a proper part of $g_{y}$. Now, take some $g_{j}\sqsubset g_{i}$ and consider a fusion of $g_{x}$ and $g_{j}$. This fusion will be a proper part of $g_{y}$, and $g_{x}$ will be a proper part of it.

This observation bears relevance to arguments by Cotnoir (2014) that purport to show that Weak Unrestricted Composition is in tension with the following principle:

- Remainder: $$y\not \sqsubseteq x \rightarrow \exists z\forall w (w\sqsubseteq z \leftrightarrow (w\sqsubseteq y\wedge \lnot Owx))$$

Cotnoir points out that the principle entails that if *y* has a proper part *x*, then there is a remainder that “has as parts all and only the non-*x*-overlapping parts of *y*.” (Cotnoir 2014 p. 657). Cotnoir claims that the “weak universalist cannot simply stipulate the remainder principle as an axiom” (Cotnoir 2014 p. 657), for she could not guarantee that all remainders can be constructed by pairwise fusion. We show in the appendix that the Remainder holds in all extensional models that are admissible according to CC. Cotnoir suggests that worlds that involve dense chains of parthood and worlds that are hunky create problems for the remainder-friendly weak universalist. To this charge we can respond with the observation that CC allows that the world is both hunky and contains dense chains of parthood, even though the Remainder principle holds in all models.

We have seen that CC does not fall prey to any of the problems that either Strong Unrestricted Composition or the different variants of Weak Unrestricted Composition that have been proposed in the literature face: Any instance of the schema is compatible with all the different mereological scenarios that have been argued in the literature to constitute genuine possibilities—i.e., gunky objects, junky worlds, hunky worlds and dense chains of parthood – and is nevertheless strong enough to determine fusion. Moreover, the instances of CC are neither trivial nor arbitrary, and constitute purely mereological rules of composition. For the contingentist, CC offers a stock of rules that she can take to hold in some worlds. For the necessitist one particular instance of CC—Countable Composition—suggest itself as the natural choice for a necessarily true rule of composition.

## Appendix

The aim of this appendix is to prove the following results for any cardinal number $\kappa$:

- Connection holds.
- If $\kappa$ is a regular cardinal, then: If *S* is a set of less than $\kappa$ many objects which each have less than $\kappa$ many non-overlapping parts, every fusion of *S* has less than $\kappa$ many non-overlapping parts.
- If $\kappa$ is a singular cardinal, then there is no model of $\hbox {CC}_\kappa$ with at least $\kappa$ many objects in its domain.
- $\hbox {CC}_{\kappa }$ is equivalent to $\hbox {CC}_{\kappa }$.
- The Remainder Principle holds in all extensional models of $\hbox {CC}_{\kappa }$.
- For every extensional model of $\hbox {CC}_{\kappa }$: Every object *x* that has a proper part is the fusion of two objects distinct from *x*.
- $\hbox {CC}_{\kappa }$ determines fusion in extensional mereology.

Unlike in the main text, where we used plural talk in order to enhance readability, we will use exclusively our official set-theoretic idiom in this appendix.

**Definitions and notation:**

- A *(mereological po) model* *M* is a pair $\langle D, \sqsubseteq \rangle$, where *D* – *the domain of* *M* – is a set of objects, and $\sqsubseteq$ – *the relation of parthood on* *D* – is a partial order.
- Two objects *x* and *y* *overlap* iff they have a common part, i.e., if there is an object *z* such that $z \sqsubseteq x \; \& \; z \sqsubseteq y$. We write “*Oxy*” for this.
- A set of objects is *overlap-free* iff no two distinct objects in the set overlap.
- Two sets *S* and *S** of objects are *overlap-equivalent* iff, for every object *x*, *x* overlaps an object in *S* iff it overlaps an object in *S**. We write “$S \equiv S$*” for this.
- An object *x* is a *fusion* of (the objects in) set *S* iff $\{x\} \equiv S$.
- (The objects in) set *S* *compose(s)* *x* iff *x* is a fusion of *S* and *S* is overlap-free.
- For $S \subseteq D$, *Par*(*S*) is the set of all the parts of objects in *S*, i.e.: $Par (S) = \{x \in D \; | \; \exists s (s \in S \; \& \; x \sqsubseteq s)\}$.
- A set of objects *S** is a *tessellation* of another set of objects *S* iff: (i) *S**$\subseteq Par(S)$, (ii) *S** is overlap-free, and (iii) *S**$\equiv S$.

### Lemma 1

*O* *is symmetric and reflexive.*

### Proof

Clear. $$\square $$

### Lemma 2

$\equiv$ *is an equivalence-relation.*

### Proof

Clear. $$\square $$

### Lemma 3

*If* $x \sqsubseteq y$ *and* *Ozx**, then* *Ozy*.

### Proof

Clear. $$\square $$

### Theorem 1

(Connection) *Every set* *S* *of objects has a tessellation.*

### Proof

Let *S* be a set of objects, and $P:=Par (S)$. By the Well-Ordering Theorem, *P* has a well-ordering. Let ‘$\le$’ denote the chosen well-ordering, and ‘<’ the associated strict order. We define a family of sets $(T_{a})_{a \in P}$ by transfinite recursion in the following way:

(Def): If there is no $x \in \bigcup _{i<a} T_{i}$ such that *a* and *x* overlap, $T_{a} = \bigcup _{i<a} T_{i} \cup \{a\}$. Otherwise, $T_{a} = \bigcup _{i<a} T_{i}$.

We now show that then, $\bigcup _{i \in P} T_{i}$ is a tessellation of *S*, i.e.: (1) $\bigcup _{i \in P} T_{i} \subseteq Par (S)$, (2) $\bigcup _{i \in P} T_{i}$ is overlap-free, (3), $\bigcup _{i \in P} T_{i} \equiv S$.

1. Clear.
2. Suppose for contradiction that $\bigcup _{i \in P} T_{i}$ is not overlap-free. Then, there are two distinct overlapping objects $x, y \in \bigcup _{i \in P} T_{i}$. Since *x* and *y* are distinct, one must be smaller. Suppose, without loss of generality, that it is *x*. Now, given the construction, *x* will be clearly the smallest $i \in P$ such that $x \in T_{i}$, and *y* the smallest $i \in P$ such that $y \in T_{i}$. Hence, $x \in T_{x} \subseteq \bigcup _{i<y} T_{i}$. Thus, given that *x* and *y* overlap, by (Def), $T_{y} = \bigcup _{i<y} T_{i}$. Since $y \in T_{y}$, it follows that there is an $i < y$ such that $y \in T_{i}$. Contradiction with the fact that *y* is the smallest $i \in P$ such that $T_{i}$ contains *y*.
3. *Left-to-right*: Let *x* be an object that overlaps some $y \in \bigcup _{i \in P} T_{i} \subseteq P = Par (S)$. Hence, by Lemma 3, *x* overlaps an object in *S*.

*Right-to-left*: Let *x* be an object that overlaps an object in *S*. Thus, there is a *y* that is part of *x* and part of an object in *S*. Hence, $y \in P$. There are two cases to consider: (i), there is a $z \in \bigcup _{i <y} T_{i}$ that overlaps *y*, (ii), there is no such *z*. In case (i), we have that $z \in \bigcup _{i \in P} T_{i}$. Since *y* is part of *x* and *y* overlaps an object in $\bigcup _{i \in P} T_{i}$, by Lemma 3, *x* overlaps an object in $\bigcup _{i \in P} T_{i}$. In case (ii), since *y* does not overlap any $z \in \bigcup _{i <y} T_{i}$, by (Def), $T_{y} = \bigcup _{i < y} T_{i} \cup \{y\}$. Hence, $y \in T_{y}$, and consequently, $y \in \bigcup _{i \in P} T_{i}$. Thus, by overlapping *y*, *x* overlaps an object in $\bigcup _{i \in P} T_{i}$. $\square$

**Definitions and notation:**

- The *cofinality* $cf (\kappa )$ of a cardinal $\kappa$ is the smallest cardinal $\lambda$ such that some union of $\lambda$ many sets of cardinality less than $\kappa$ has the cardinality $\kappa$.
- A cardinal $\kappa$ is *regular* iff it is has a cofinality that equals $\kappa$. A cardinal $\kappa$ is *singular* iff it is not regular.

### Theorem 2

*Let* $\kappa$ *be some regular cardinal. Let* *S* *be a set of less than* $\kappa$ *many objects such that, for every* $s \in S$ *and every overlap-free* $A_s \subseteq Par(\{s\})$*,* $|A_s | < \kappa$. *Then: If* *S* *has a fusion* *o**, for every overlap-free* $B\subseteq Par(\{o\})$*,* $|B| < \kappa$.

### Proof

Let *S* and $\kappa$ be as specified in Theorem 2, and let *o* be a fusion of *S*. Let $B\subseteq Par(\{o\})$ be overlap-free but otherwise arbitrary. For every $s \in S$, let $P_s:= Par (\{s\}) \cap Par(B)$. By Theorem 1, every $P_s$ has a tessellation $T_s$. Now, consider $T:= \bigcup _{s \in S} T_s$. We show that (1), $|B | \le |T|$ and, (2), $|T | < \kappa$. Then, it directly follows that $|B | < \kappa$.

1. By the definition of *T* and the transitivity of parthood, every object in *T* is part of an object in *B*. Moreover, given that *B* is overlap-free, no object in *T* is part of more than one object in *B*. Hence, there is a unique function $f: T\rightarrow B$ that maps every element of *T* to the element of *B* that it is part of. What remains to be shown in order to prove that $|B | \le |T|$ is that *f* is surjective – which is equivalent to the fact that, for every object in *B*, there is an object in *T* that is part of it. Now, let $b \in B$ be arbitrary. Then, since $B \subseteq Par (\{o\})$, *b* overlaps *o*, and since $o \equiv S$, *b* overlaps some object $s \in S$. Let *c* be a common part of *b* and *s*. Then, by the definition of $P_s$, $c \in P_s$. Given that $P_s \equiv T_s$, there is a $d \in T_s$ that overlaps *c*, and thus, by Lemma 3, also *b*. Since every object in $T_s$ is part of an object in *B*, *d* is part of some $e \in B$. Now, given that *d* and *b* overlap, and *d* is part of *e*, by Lemma 3, *b* and *e* overlap. Since *B* is overlap-free, and *b* and *e* are both elements of *B*, it follows that *b* and *e* must be identical. And thus, since *d* is part of *e*, it is part of *b*. Hence, there is an object in $T_s$, and thus in *T*, that is part of *b*, as we wanted to show.
2. Given that, for every *s*, all the elements of $T_s$ are parts of *s* and $T_s$ is overlap-free, by our assumption, $|T_s| < \kappa$. Assume for reductio that $|T | \ge \kappa$. Then, there is a $R \subseteq T$ with $|R| = \kappa$. Now, $R = R \cap T = R \cap \bigcup _{s \in S} T_s = \bigcup _{s \in S} (R \cap T_s)$. And for every $s \in S$, $|R \cap T_s| \le |T_s| < \kappa$. *R* is thus a set of cardinality $\kappa$ that is the union of |*S*| many sets of cardinality less than $\kappa$. Hence, $cof(\kappa ) \le |S|$. Since, by assumption, $|S| < \kappa$, it follows that $cof(\kappa ) < \kappa$. Contradiction with the assumption that $\kappa$ is regular. $\square$

**Definitions and notation:**

Where $\kappa$ is some cardinal number:

$\hbox {CC}_{\kappa }$: *S* composes an object iff *S* is overlap-free, non-empty and of cardinality smaller than $\kappa$.

### Theorem 3

*Let* $\kappa$ *be a singular cardinal. Then: There is no model of* $CC_{\kappa }$ *whose domain contains at least* $\kappa$ *many non-overlapping objects.*

### Proof

Let $\kappa$ be a singular cardinal. Assume for reductio that there is a model *M* of $CC_{\kappa }$ whose domain contains at least $\kappa$ many non-overlapping objects.

Now, note first that, (i), the only non-zero cardinal that has no cofinality is 1, and that (ii), the cofinality of a cardinal $\kappa$ is never larger than $\kappa$ itself. This can be seen by noting that every set is the union of the singletons of its members. Hence, if $\kappa > 1$, every set of cardinality $\kappa$ is the union of $\kappa$ many sets of cardinality smaller than $\kappa$. This guarantees that, if some cardinal $\kappa > 1$ has a cofinality, this cofinality is at most $\kappa$.

Moreover, since the common ordering of the cardinals is a well-ordering, if there is a cardinal $\lambda$ such that $\kappa$ is the union of $\lambda$ many sets of cardinality less than $\kappa$, there must be a smallest one. That is, $\kappa$ must have a cofinality. 1, by contrast, has no cofinality, since the only set of cardinality less than 1 is the empty set, and no set with one element can be build by unions of the empty set. Now, since we have assumed that $\kappa$ is singular, either (a*) has no cofinality or (b*) has a cofinality $\lambda \ne \kappa$. By (i) and (ii), these two options amount to the following: (a) $\kappa = 1$, or, (b) $\kappa > 1$ has a cofinality $\lambda$ with $\lambda < \kappa$.

- Suppose for contradiction that there is a model of $CC_{1}$ whose domain contains at least one object, say *o*. By the definition of composition, *o* composes itself. And by the left-to-right direction of $CC_1$, it follows that *o*’s singleton contains less than 1 element. Contradiction.
- Since $\lambda$ is the cofinality of $\kappa$, there is a set *S* with $|S| = \kappa$ and a family of sets $(S_i)_{i \in I}$ such that (i) $S = \bigcup _{i \in I} S_i$, (ii) $|I| = \lambda$, and, (iii) for any $i \in I$, $|S_i | < \kappa$. Since, by assumption, $\kappa > 1$, $S \ne \emptyset$. Let $(a_{s})_{s \in S}$ be a family of overlap-free distinct objects in the domain of *M*. That there is such a family is guaranteed by the fact that $|S|= \kappa$ and that, by our assumption, the domain of *M* contains $\kappa$ many non-overlapping objects. Now, let $\le$ be a well-order on *I*, and < the associated strict order. For any $i \in I$, let $T_i = S_i \setminus \bigcup _{j < i} S_j$. Then, *S* is the disjoint union of the $T_i$, and thus, $\{a_s \, | \, s \in S\}$ the disjoint union of the sets $\{a_s \, | \, s \in T_i\}$. Now, for every $i \in I$, $|\{ a_s \, | \, s \in T_i\}| = |T_i| \le |S_i|< \kappa$. Since the $a_i$ do not pairwise overlap, by the right-to-left direction of $CC_\kappa$, it follows that, for every $i \in I$ for which $T_i \ne \emptyset$, the objects in $\{ a_s \, | \, s \in T_i\}$ compose an object, say $b_i$. There are as many $b_i$ as non-empty $T_i$. So there are no more $b_i$ than $|I| = \lambda < \kappa$. Moreover, since the sets $\{a_s \, | \, s \in T_i\}$ are disjoint and their union $\{a_s \, | \, s \in S\}$ is overlap-free, none of the $b_i$ pairwise overlap. Finally, since $S \ne \emptyset$ and $S = \bigcup _{i \in I} T_i$, at least one of the $T_i$ is non-empty. Hence, there is at least one $b_i$. By the right-to-left direction of $CC_{\kappa }$, it follows that the $b_i$ compose an object, say *b*. *b* has all the objects in $\{a_s \, | \, s \in S\}$, and thus $\kappa$ many non-overlapping objects as a part. Contradiction with the left-to-right direction of $CC_\kappa$. $\square$

**Definitions and notation:**

For any cardinal number $\kappa$:

$\hbox {CC}_{\kappa }$*: *S* has a fusion iff there is a non-empty tessellation of *S* of cardinality smaller than $\kappa$.

### Lemma 4

*If* *S* *has a fusion* *x**,* $S \ne \emptyset$.

### Proof

Clear, given that *x* overlaps itself. $\square$

### Theorem 4

(Equivalence) *For every cardinal* $\kappa$*,* $\hbox {CC}_{\kappa }$ *and* $\hbox {CC}_{\kappa }$* *are equivalent, i.e., have the same models.*

### Proof

$\hbox {CC}_{\kappa } \Rightarrow \hbox {CC}_{\kappa }$*: Suppose that $\hbox {CC}_{\kappa }$ holds for some cardinal $\kappa$.

Suppose that *S* has a fusion, say *x*. By Theorem 1, there is a tessellation *T* of *S*. Since $T \equiv S$ and $S \equiv \{x\}$, by the transitivity of $\equiv$, $T \equiv \{x\}$. Since, moreover, *T* is overlap-free, *T* composes *x*. By $\hbox {CC}_{\kappa }$, it follows that *T* has less than $\kappa$ many members, and by Lemma 4 that *T* is non-empty. Suppose that *T* is a non-empty tessellation of *S* with less than $\kappa$ many members. Then, by $\hbox {CC}_{\kappa }$, *T* composes, and thus fuses, an object. Since $T \equiv S$, by the transitivity of $\equiv$, this object is also the fusion of *S*.

$\hbox {CC}_{\kappa }* \Rightarrow \hbox {CC}_{\kappa }$: Suppose that $\hbox {CC}_{\kappa }$* holds for some cardinal $\kappa$.

Suppose that *S* composes an object. Hence, *S* is overlap-free, and, by Lemma 4, non-empty. What remains to be shown is that *S* has less than $\kappa$ many members. Since *S* composes an object, by $\hbox {CC}_{\kappa }$*, there is a tessellation *T* of *S* that has less than $\kappa$ many members. Now, let $s \in S$ be arbitrary. Given that $T \equiv S$ and *Oss*, there must be some $t \in T$ such that *Ots*. Since $T \subseteq Par(S)$, there is an $s' \in S$ such that $t \sqsubseteq s'$. Hence, by Lemma 3, $Os's$. Now, since *S* composes an object, *S* is overlap-free, and thus, given that $Os's$, it follows that $s = s'$. Thus, $t \sqsubseteq s$. Since *s* was arbitrarily chosen, it follows that, for very $s \in S$, there is a $t \in T$ such that $t \sqsubseteq s$. Moreover, since *S* is overlap-free, no $t \in T$ is part of two distinct objects in *S*. So there is a surjective function $f : T \rightarrow S$ that maps every $t \in T$ to the unique $s \in S$ such that $t \sqsubseteq s$. It follows that there are no more objects in *S* than in *T*, and thus, that *S* has less than $\kappa$ many members.

Suppose that *S* is non-empty, overlap-free and has less than $\kappa$ many members. Given that $S \equiv S$ and $S \subseteq Par(S)$, *S* is a non-empty, tessellation of itself that has less than $\kappa$ many members. Hence, by $\hbox {CC}_{\kappa }$*, *S* has a fusion. Since, moreover, *S* is overlap-free, *S* composes this object. $\square$

**Definitions and notation:**

- A model is *extensional* iff it obeys the following principle of *Strong Supplementation*: $\lnot y \sqsubseteq x \rightarrow \exists z (z\sqsubseteq y \; \& \; \lnot Ozx)$.

### Theorem 5

(Remainder) *Let* *M* *be an extensional model that is admissible with regard to* $\hbox {CC}_{\kappa }$ *for some cardinal* $\kappa$. *Let* $x, y \in D$ *with* $\lnot y \sqsubseteq x$. *Then: There is a* $z \in D$ *that is a remainder of* *x* *from* *y**, i.e., such that:* $\forall w \; (w \sqsubseteq z \leftrightarrow (w \sqsubseteq y \; \& \; \lnot Owx))$.

### Lemma 5

*For any model* *M* *of extensional mereology, and objects* $x, y \in D$*: If* $P^{-} := \{w \; | \; w \in Par (\{y\}) \; \& \; \lnot Owx\}$ *has a fusion, this fusion is a remainder of* *x* *from* *y*.

### Proof

Assume that $P^{-}$ has a fusion, say *z*.

Let $w \sqsubseteq z$. We show that then, (a) $w \sqsubseteq y$, and (b) $\lnot Owx$.

- Suppose for contradiction that $w \not \sqsubseteq y$. Then, by Strong Supplementation, there is a *v* such that $v \sqsubseteq w$ and $\lnot Ovy$. Since $w \sqsubseteq z$, by the transitivity of parthood, $v \sqsubseteq z$. Thus, *Ovz*. Then, given that *z* is a fusion of $P^{-}$, there is a $u \in P^{-}$ such that *Ovu*. By the definition of $P^{-}$, it follows that $u \sqsubseteq y$. Since *Ovu* and $u \sqsubseteq y$, by Lemma 3, *Ovy*. Contradiction.
- Suppose for contradiction that *Owx*. Then, since $w \sqsubseteq z$, by Lemma 3, *Ozx*. Given that *z* is the fusion of $P^{-}$, it follows that there is a $v \in P^{-}$ such that *Ovx*. However, by the definition of $P^{-}$, no object in $P^{-}$ overlaps *x*. Contradiction.

Let $w \sqsubseteq y$ be such that $\lnot Owx$, i.e., such that $w \in P^{-}$. We show that then, $w \sqsubseteq z$.

Suppose for contradiction that $w \not \sqsubseteq z$. Then, by Strong Supplementation, there is a $v \sqsubseteq w$ such that $\lnot Ovz$. Thus, *Ovw*, but $\lnot Ovz$. Contradiction with the fact that $w \in P^{-}$ and *z* is a fusion of $P^{-}$. $\square$

### Proof of Theorem 5

Let *M* be an extensional model of $\hbox {CC}_{\kappa }$ and $x, y \in D$ with $y \not \sqsubseteq x$. Let $P := Par (y)$. Let $P^{-} := \{w \; | \; w \in Par (\{y\}) \; \& \; \lnot Owx\}$ and $P^{+} := \{w \; | \; w \in Par (\{y\}) \; \& \; Owx\}$. Clearly, $P^{-}$ and $P^{+}$ are disjoint and have *P* as their union. Moreover, given that $\lnot y \sqsubseteq x$, by Strong Supplementation, $P^{-} \ne \emptyset$. We well-order both $P^{-}$ and $P^{+}$. Let $\le ^{-}$ and $\le ^{+}$ be the chosen well-orderings on $P^{-}$ and $P^{+}$, respectively. We next build the sum of these two well-orderings, i.e., the ordering $\le ^{-} \cup \le ^{+} \cup \; \{<x, y> | \; x \in P^{-}, y \in P^{+}\} \; =: \; \le$. (That is, $\le$ is an ordering in which every element of $P^{-}$ precedes every element of $P^{+}$, and pairs of elements from $P^{-}$ or $P^{+}$ retain their original order.) Given that the sum of two well-orderings on disjoint sets is a well-ordering on the union of the sets, $\le$ is a well-ordering on *P*.

We then define two families of sets $(T_{a})_{a \in P}$ and $(T^{-}_{a})_{a \in P^{-}}$ by transfinite recursion in the same way as in the proof for Theorem 1. (That is: If there is no $x \in \bigcup _{i<a} T_{i}$ such that *a* and *x* overlap, $T_{a} = \bigcup _{i<a} T_{i} \cup \{a\}$. Otherwise, $T_{a} = \bigcup _{i<a} T_{i}$. And in parallel for $(T^{-}_{a})_{a \in P^{-}}$.)

We now show that $\bigcup _{i \in P^{-}} T^{-}_{i}$ (a) is a tessellation of $P^{-}$, (b) is non-empty, and (c) has less than $\kappa$ many members. Then, by $\hbox {CC}_{\kappa }$*, it follows that $P^{-}$ has a fusion, and, by Lemma 5, that this fusion is a remainder of *y* from *x*.

- First, note that $P^{-} = Par (P^{-})$: Clearly, $P^{-} \subseteq Par (P^{-})$. To see that also $Par (P^{-}) \subseteq P^{-}$, let $w \in Par (P^{-})$ be arbitrary. Then, by the transitivity of parthood, it follows that $w \in Par (\{y\})$, and by Lemma 3, that $\lnot Owx$. Thus, $w \in P^{-}$. Now, given that $P^{-} = Par (P^{-})$, we can apply the same reasoning as in the proof of Theorem 1 to $(T^{-}_{a})_{a \in P^{-}}$, thus getting the result that $\bigcup _{i \in P^{-}} T^{-}_{i}$ is a tessellation of $P^{-}$.
- Clear, given that $P^{-} \ne \emptyset$.
- Given that $\le ^{-}$ is an initial sequence of $\le$, our constructions for $(T_{a})_{a \in P}$ and $(T^{-}_{a})_{a \in P^{-}}$ will be clearly such that, for every $a \in P^{-}$, $T_{a} = T^{-}_{a}$. Hence, $\bigcup _{i \in P^{-}} T^{-}_{i} = \bigcup _{i \in P^{-}} T_{i} \subseteq \bigcup _{i \in P} T_{i}$. Now, applying the same reasoning as in the proof for Theorem 1 to $\bigcup _{i \in P} T_{i}$ yields that $\bigcup _{i \in P} T_{i}$ is a tessellation of $\{y\}$. Hence, the objects in $\bigcup _{i \in P} T_{i}$ compose *y*, and by $\hbox {CC}_{\kappa }$, $\bigcup _{i \in P} T_{i}$ has less than $\kappa$ many members. And thus, also its subset $\bigcup _{i \in P^{-}} T^{-}_{i}$ has less than $\kappa$ many members. $\square$

### Theorem 6

(NCC Implies Weak Unrestricted Splitting) *Let* *M* *be an extensional model that is admissible with regard to* $\hbox {CC}_{\kappa }$ *for some cardinal* $\kappa$. *Let* *x* *be an object that has a proper part. Then:* *x* *is the fusion of two objects distinct from* *x*.

### Proof

Let *M* be an extensional model that is admissible with regard to $\hbox {CC}_{\kappa }$ for some cardinal $\kappa$. Let *x* be an object that has a proper part, say *y*. Then, by Theorem 5, there is a remainder of *y* from *x*, say *y**. Now, since *y* overlaps itself and *y** is a reminder of *y* from *x*, *y* is not a part of *y**. Given that *y* is a part of *x*, it follows that *y** and *x* are distinct. And since *y* is a proper part of *x*, *y* and *x* are also distinct. Thus, in order to prove Theorem 6, it suffices to show that *x* is the fusion of *y* and *y**, that is, that an object *z* overlaps *x* iff it overlaps *y* or *y**.

Let *z* overlap *x*. We need to show that *z* overlaps *y* or *y**. Suppose that *z* does not overlap *y*. Now, since *z* overlaps *x*, there is a *z** that is part of both *z* and *x*. Then, since *z* does not overlap *y*, *z** does not either. From this and the fact that *z** is part of *x* and *y** a remainder of *y* from *x*, it follows that *z** is part of *y**. Hence, *z* overlaps *y**.

Let *z* overlap *y* or *y**. Given that *y* is a proper part of *x*, if *z* overlaps *y*, by Lemma 3, it overlaps *x*. Now, suppose that *z* overlaps *y**. Then, there is a *z** that is part of both *z* and *y**. Since *y** is a remainder of *y* from *x*, it follows that *z** is part of *x*. Hence, *z* overlaps *x*. $\square$

**Definitions and notation:**

- A model $$M_{1} $$ is a *submodel* of another (possibly identical) model $$M_{2}$$ iff $$D_{1} \subseteq D_{2}$$ and $$\sqsubseteq _{1} = \sqsubseteq _{2} \cap \, (D_{1} \times D_{1})$$, i.e., $$M_{1}$$’s relation of parthood is $$M_{2}$$’s restricted to $$D_{1}$$.
- A model $M_{1}$ is a *base* for another model $M_{2}$ iff $M_{1}$ is a submodel of $M_{2}$ such that, for every $x \in D_{2}$, there is a $S \subseteq D_{1}$ such that *x* is the fusion (with regard to $M_{2}$) of *S*.
- A rule of composition *determines fusion* iff, for all models $M_{1}, M_{2}$ that are admissible with regard to this rule: If $M_{1}$ is a base for $M_{2}$, then $M_{1} = M_{2}$.

The aim of the following is to prove that, for any cardinal $\kappa$, in extensional mereology, $\hbox {CC}_{\kappa }$ determines fusion. For this, we will first have to prove a number of auxiliary results, before we can proceed to the proof of the theorem.

### Lemma 6

*In every extensional model, if* *x* *and* *y* *are both fusions of the same set, then* $x = y$.

### Proof

Let *x* and *y* be fusions of *S*. That is, we have that $\{x\} \equiv S \equiv \{y\}$, and thus, by the transitivity of $\equiv$, that $\{x\} \equiv \{y\}$. Now, suppose for contradiction that $x \ne y$. Then, by the antisymmetry of parthood, either (a) $x \not \sqsubseteq y$, or (b), $y \not \sqsubseteq x$ (or both). Suppose, without loss of generality, that (a). Then, by Strong Supplementation, there is a $z \sqsubseteq x$ such that $\lnot Ozy$. Thus, *Ozx* but $\lnot Ozy$. Contradiction with the fact that $\{x\} \equiv \{y\}$. $\square$

### Remark

Due to Lemma 6, we can in the following speak of ‘the fusion’ rather than ‘a fusion’ of a set of objects.

### Lemma 7

*In every extensional model, if* *x* *is the fusion of* *S**, all the objects in* *S* *are parts of* *x*.

### Proof

Assume that *x* is the fusion of *S*. Suppose for contradiction that there is a $y \in S$ such that $y \not \sqsubseteq x$. Then, by Strong Supplementation, there is a $z \sqsubseteq y$ such that $\lnot Ozx$. Since $z \sqsubseteq y$, *Ozy*. Hence, *Ozy* but $\lnot Ozx$. Contradiction with the assumption that *x* is the fusion of *S*. $\square$

### Remark

In difference to the proofs thus far, in what follows, more than one model will be at stake at once. To specify whether the relevant mereological notions concern model $M_{1}$ or $M_{2}$, we will thus make use of the indexes ‘1’ and ‘2’.

### Lemma 8

*Let* $M_{1}$ *be a base for* $M_{2}$. *Then:* $x, y \in D_{2}$ *have a common part in* $D_{1}$ *iff they have a common part in* $D_{2}$.

### Proof

Let $x, y \in D_{2}$ be arbitrary.

Let *x*, *y* have a common part in $D_{1}$, say *z*. Then, given that $D_{1} \subseteq D_{2}$ and that $D_{1}$-parthood is $D_{2}$-parthood restricted to $D_{1}$, *x* is a common part in $D_{2}$ of *x* and *y*.

Let $x, y \in D_{2}$ have a common part in $D_{2}$, say *z*. Then, since $M_{1}$ is a base for $M_{2}$, there is a $S\subseteq D_{1}$ such that *z* is the fusion_2_of *S*. By Lemma 7, all the objects in *S* are parts of *z*, and thus, by the transitivity of parthood, parts of both *y* and *x*. Moreover, by Lemma 4, $S \ne \emptyset$. Hence, *y* and *x* have a common part in *S*, and thus in $D_{1}$. $\square$

### Lemma 9

*Let* $M_{1}, M_{2}$ *be extensional models with* $M_{1}$ *as a base for* $M_{2}$. *Then:* $S \subseteq D_{1}$ *is overlap*_2_-*free iff* *S* *is overlap*_1_-*free.*

### Proof

Follows directly from Lemma 8. $\square$

### Lemma 10

*Let* $M_{1}, M_{2}$ *be extensional models with* $M_{1}$ *as a base for* $M_{2}$. *Then: For all* $S, U \subseteq D_{1}$, $S \equiv _{2}U$ *iff* $S \equiv _{1}U$.

### Proof

Let $S, U \subseteq D_{1}$ with $S \equiv _{2} U$. Let $x \in D_{1}$ overlap_1_some $s \in S$.

By Lemma 8, *x* and *s* overlap_2_. Since $S \equiv _{2} U$, it follows that there is an $u \in U$ such that *x* overlaps_2_ *u*. Thus, by Lemma 8, *x* and *u* have a common part in $D_{1}$, that is, *x* overlaps_1_a member of *U*.

The case that $x \in D_{1}$ overlaps_1_an $u \in U$ is analogous.

Let $S, U \subseteq D_{1}$ with $S \equiv _{1} U$. Let $x \in D_{2}$ overlap_2_some $s \in S$. By Lemma 8, *x* and *s* have a common part in $D_{1}$, say *y*. Since $S\equiv _{1}U$, there is an $u\in U$ such that *y* overlaps_1_ *u*. By Lemma 8, *y* overlaps_2_ *u*. By Lemma 3, it follows that *x* overlaps_2_ *u*.

The case that $x \in D_{2}$ overlaps_2_an $u \in U$ is analogous. $\square$

### Theorem 7

(Determination of Fusion) *For any* $\kappa$*,* $\hbox {CC}_{\kappa }$ *determines fusion in extensional mereology.*

### Proof

Let $M_{1}$ and $M_{2}$ be models of extensional mereology that are admissible with respect to $\hbox {CC}_{\kappa }$ for some cardinal $\kappa$, such that $M_{1}$ is a base for $M_{2}$. To prove Theorem 6, we have to show that $D_{1} = D_{2}$. For this, we consider some arbitrary $x \in D_{2}$ and show that $x \in D_{1}$. Then, given that $D_{1} \subseteq D_{2}$, it follows that $D_{1} = D_{2}$.

Since $x \in D_{2}$ and $M_{1}$ is a base for $M_{2}$, there is a $S \subseteq D_{1}$ such that *x* is the fusion_2_ of *S*, i.e., such that $S \equiv _{2} \{x\}$. We now apply Theorem 1 to *S* within the context of $M_{1}$. This gives us that there is an overlap_1_-free set $S' \subseteq Par_{1}(S) \subseteq D_{1}$ with $S' \equiv _{1} S$. Now, since $S \equiv _{1} S'$, by Lemma 10, $S' \equiv _{2} S$. And given that $S \equiv _{2} \{x\}$, by the transitivity of $\equiv _{2}$, $S' \equiv _{2} \{x\}$. Moreover, since $S'$ is overlap_1_-free, by Lemma 9, $S'$ is overlap_2_-free. Hence, the objects in $S'$ compose_2_ *x*.

By $\hbox {CC}_{\kappa }$, it follows that $S'$ has less than $\kappa$ many members, and by Lemma 4 that $S \ne \emptyset$. Moreover, $S'$ is overlap_1_-free. Hence, by $\hbox {CC}_{\kappa }$, the objects in $S'$ compose_1_an object, say *y*. Thus, $S' \equiv _{1} \{y\}$. By Lemma 10, it follows that $S' \equiv _{2} \{y\}$, i.e., that $S'$ has *y* as its fusion_2_. Since *x* and *y* are fusions_2_of $S'$ and $M_{2}$ is extensional, by Lemma 6, $x=y$. Thus, given that $y \in D_{1}$, $x\in D_{1}$. $\square$

## Footnotes

1 A dissenter with respect to this thesis is Markosian (1998a).

2 In the main text, we formulate matters in terms of pluralities in order to enhance readability. Our official idiom is set-theoretic, however. We assume ZFC including, in particular, the axiom of choice. Whether discussions of the special composition question should be approached in terms of plural logic, first-order logic with set theory, first-order logic using sentence schemas, or in higher-order logic is a contentious question that we cannot hope to address in this paper (cf. Hovda 2009). We leave the question of which considerations of this paper could be redeemed within another setup for another time.

3 Throughout this paper, we will use “$\sqsubseteq$” to denote parthood and “$\sqsubset$” to denote proper parthood. As it is standard, we define overlap as follows: $Oxy\equiv _{def}\exists z (z\sqsubseteq x\wedge z\sqsubseteq y)$. With this notation and definition in place, Strong Supplementation can be expressed as follows: $\lnot x\sqsubseteq y\rightarrow \exists z(z\sqsubseteq x\wedge \lnot Ozy)$.

4 This definition of fusion is proposed as “the natural compromise” for those who do not wish to commit to the principle of Strong Supplementation by Achille Varzi in Varzi (2019). In extensional mereology, it is equivalent to the alternative definition that *y* is a fusion of the *xx* iff every one of the *xx* is a part of *y* and every part of *y* is overlapped by one of the *xx* (cf. Varzi 2019). Note also that, in extensional mereology, fusion is unique (cf. Lemma 6 in the appendix) and we can thus indeed speak of *the* fusion of the *xx*.

5 The *xx* are pairwise non-overlapping iff for every *x* and every $x'$ that are distinct and both among the *xx*, there is no *z* that is a part of *x* and a part of $x'.$

6 We assume here that there is no empty plurality, i.e., a plurality that has no objects among it. If the empty plurality were admitted, the talk of “pluralities” here and in what follows would have to be replaced by talk of “non-empty pluralities”. Moreover, given our set-theoretic approach, we presuppose that every plurality of objects is “set-sized”, i.e., that there is a set of all the objects among the plurality.

7 See Bohn (2009a) for a defence of the possibility of junky worlds.

8 Given that proper parthood is asymmetric, Weak Supplementation follows from Strong Supplementation (cf. Cotnoir 2010).

9 See Contessa (2012) and Smith (2019) for a recent defence.

10 See also Bohn (2009a) p. 30 for a similar point against this candidate rule.

11 Let $x_{1},x_{2},\ldots$ be such an infinitely descending chain. According to Weak Supplementation, there is a proper part of $x_{1}$ that does not overlap $x_{2}$ , say $x_{11}$. Likewise, there is a proper part of $x_{2}$ that does not overlap $x_{3}$ , say $x_{22}$, and so on. Now, $x_{11}$ and $x_{22}$ do not overlap, since $x_{11}$ does not overlap with $x_{2}$ and $x_{22}$ is a part of $x_{2}$. And given the transitivity of parthood, $x_{11}, x_{22},\ldots$ are proper parts of $x_{1}$. Thus, the sequence $x_{11}, x_{22},\ldots$ forms an infinite plurality of non-overlapping proper parts of $x_{1}$.

12 Smith writes that the *xx* have a fusion “when and only when either there is only one *x* [among the *xx*] or the *xx* can be coherently described as constituting two objects each of which is consistent with weak mereological universalism.”(Smith 2019). It is not entirely clear to us what Smith exactly means with some objects being such that one can “coherently” describe them as “constituting two objects each of which is consistent with weak mereological universalism”, but we take the combination of Weak Unrestricted Composition and Weak Unrestricted Splitting to be the natural candidate for a precisification of her claims.

13 To see this, recall that, if $y_{1}$ and $y_{2}$ are jointly overlap-equivalent to the *xx*, then $y_{1}$ and $y_{2}$ have a fusion iff the *xx* have, and their respective fusions are identical.

14 To see that $w_{a}$ validates Weak Unrestricted Splitting, note again that every object *x* in $w_{a}$ is the fusion of the members of a finite plurality of simples $s_{1},... s_{n}$. Thus, *x* is e.g. the fusion of $s_{1}$ with the fusion of $s_{2},...s_{n}$.

15 What we mean by saying that some object $x \in D_{2}$ is the fusion of some plurality *yy* of objects *with regard to* $M_{2}$ is that, for every object *z* *in* $D_{2}$, *z* and *x* have a common part *in* $D_{2}$ iff *z* and one of the *yy* have a common part *in* $D_{2}$.

16 Often, the terminology of regular versus singular cardinals is exclusively applied to infinite cardinals. It should be also noted that different available definitions of cofinality that lead to the same results in the realm of the infinite cardinals lead to different results when applied to finite cardinals.

17 To construct two sets of cardinality less than *n* (for $n >2$) such that their union has cardinality *n*, let e.g. the first set be the set of the even and the other set be the set of the odd cardinals smaller than *n*.

18 One might think of the following alternative rule schema CC’ and wonder how it relates to the rule schema CC proposed here: CC’: The *xx* compose an object iff the *xx* are pairwise non-overlapping and of cardinality smaller than or equal to $\kappa$. Now, given that a cardinality is smaller-or-equal to $\kappa$ iff it is smaller than $\kappa + 1$, CC’_κ_ is equivalent to $\hbox {CC}_{\kappa + 1}$. That is, every instance of CC’ corresponds to an instance of CC. Moreover, since every infinite successor cardinal is regular, every instance of CC’ for an infinite $\kappa$ corresponds to an instance of CC for a regular $\kappa$. However, the converse fails: If $\kappa$ is a regular limit cardinal, then there is no instance of CC’ that corresponds to CC’_κ_. In particular, no instance of CC’ corresponds to $\hbox {CC}_{\aleph _0}$, the rule according to which every finite plurality has a fusion. And since $\hbox {CC}_{\aleph _0}$ is (as we will show later in the paper), the only instance of CC that is compatible with world $w_a$, CC but not CC’ is compatible with $w_a$. For this reason, we believe that CC should be preferred over CC’. We thank a reviewer for discussion.

19 That there is no largest regular cardinal follows from the fact that every successor cardinal is regular.

20 A line of reasoning similar to the one employed here seems to stand in the background of the discussion of gunk and Weak Unrestricted Composition in Smith (2019) §2, where Smith describes a gunky object that at every “level” can “be taken to be properly composed of two objects” (p. 48). We acknowledge that the case of gunk is not analogous to every way of how the creation of a fusion of countably infinitely many simples can be conceptualised. To see this, consider the case of the quick fuser who starts with fusing simples $s_1$ and $s_2$, then adds $s_{3}$ and manages to fuse all $s_i$ for $i\in {\mathbb {N}}$ in a finite amount of time. The quick fuser might do so by accelerating: They fuse $s_1$ and $s_2$ in half an hour, add $s_{3}$ in the next quarter of an hour, add $s_4$ in the following eighth of an hour and so on *ad infinitum*. If we assume that this is possible (a question that points to issues concerning the philosophy of supertasks we cannot tackle in the context of this paper), the quick fuser can complete the fusion of countably infinitely many simples in an hours time. Such a story arguably cannot be told for gunky objects, for they do not have a simple part the quick fuser could start with. We thank a referee for discussion.

21 Philosophers worried by this result who wish to uphold Countable Composition for objects other than spacetime points could adopt a pluralist take on rules of composition, thus maintaining that which rules of composition apply to objects is kind-dependent (cf. van Inwagen 1990), and that, in particular, separate rules of composition apply to spacetime points.

22 In this context it should be noted, though, that a number of philosophers deny (ii) because it is incompatible with extended simples in a pointy space (see e.g. van Inwagen 1990; Markosian 1998b).

23 Here *i* ranges over all finite sequences of the digits “1” and “2”, including the empty sequence consisting of no digit as a limit case.

24 See e.g. Bohn (2018) and Cotnoir (2014) for uses of the notion of hunk.
